# The shoulder girdle of early chondrichthyans grew by skeletal remodelling

**DOI:** 10.1098/rsbl.2025.0411

**Published:** 2025-09-24

**Authors:** Plamen S. Andreev, Min Zhu, Lars Brakenhoff, Qiang Li, Wenjin Zhao, Lijian Peng, Federica Marone, Richard P. Dearden, Martin Rücklin

**Affiliations:** ^1^ Research Center of Natural History and Culture, Qujing Normal University, Qujing, China; ^2^ Department of Understanding Evolution, Naturalis Biodiversity Center, Leiden, Netherlands; ^3^ School of Geography, Earth and Environmental Sciences, University of Birmingham, Birmingham, UK; ^4^ Key CAS Laboratory of Vertebrate Evolution and Human Origins, Institute of Vertebrate Paleontology and Paleoanthropology, Chinese Academy of Sciences, China, Beijing, China; ^5^ CAS Center for Excellence in Life and Paleoenvironment, Beijing, China; ^6^ College of Earth and Planetary Sciences, University of Chinese Academy of Sciences, Beijing, China; ^7^ Swiss Light Source, Paul Scherrer Institut, Villigen, Switzerland; ^8^ Institute of Biology, University of Leiden, Leiden, Netherlands

**Keywords:** bone, resorption, Silurian, dermal skeleton, dentine, jawed vertebrates, fin spine

## Abstract

A distinct shoulder region, defined by endoskeletal and dermal girdles and associated pectoral musculature, is a major evolutionary adaptation of jawed vertebrates. In teleost model species, the large (macromeric) pectoral dermal bones can be derived from multiple embryonic tissues, identifying the shoulder of osteichthyans as a developmentally complex area at the head–trunk boundary. The absence of bone in living chondrichthyans makes Palaeozoic stem groups capable of dermal ossification key to understanding the underpinnings of skeletal growth in the shoulder of crown gnathostomes (osteichthyans and chondrichthyans). Here, using synchrotron X-ray tomography we demonstrate that individual pectoral plates in the oldest unequivocal jawed vertebrate, the Silurian (c. 439 Mya) chondrichthyan *Fanjingshania renovata*, develop from five separate growth centres. These centres correspond to pectoral bony spines that fuse neighbouring dermal scales into a pinnal plate and their expansion is accompanied by cyclical resorption and remodelling of bone and dentine. Our phylogenetic analyses support an interpretation of these processes as crown and stem gnathostome characters that co-occur only in the shoulder girdle of stem chondrichthyans. The systematic hard tissue remodelling in *Fanjingshania* reveals an unexpected growth dynamic within chondrichthyans that relates to the formation of a macromeric skeleton through integration of modular elements.

## 1. Introduction

**Figure 1.**
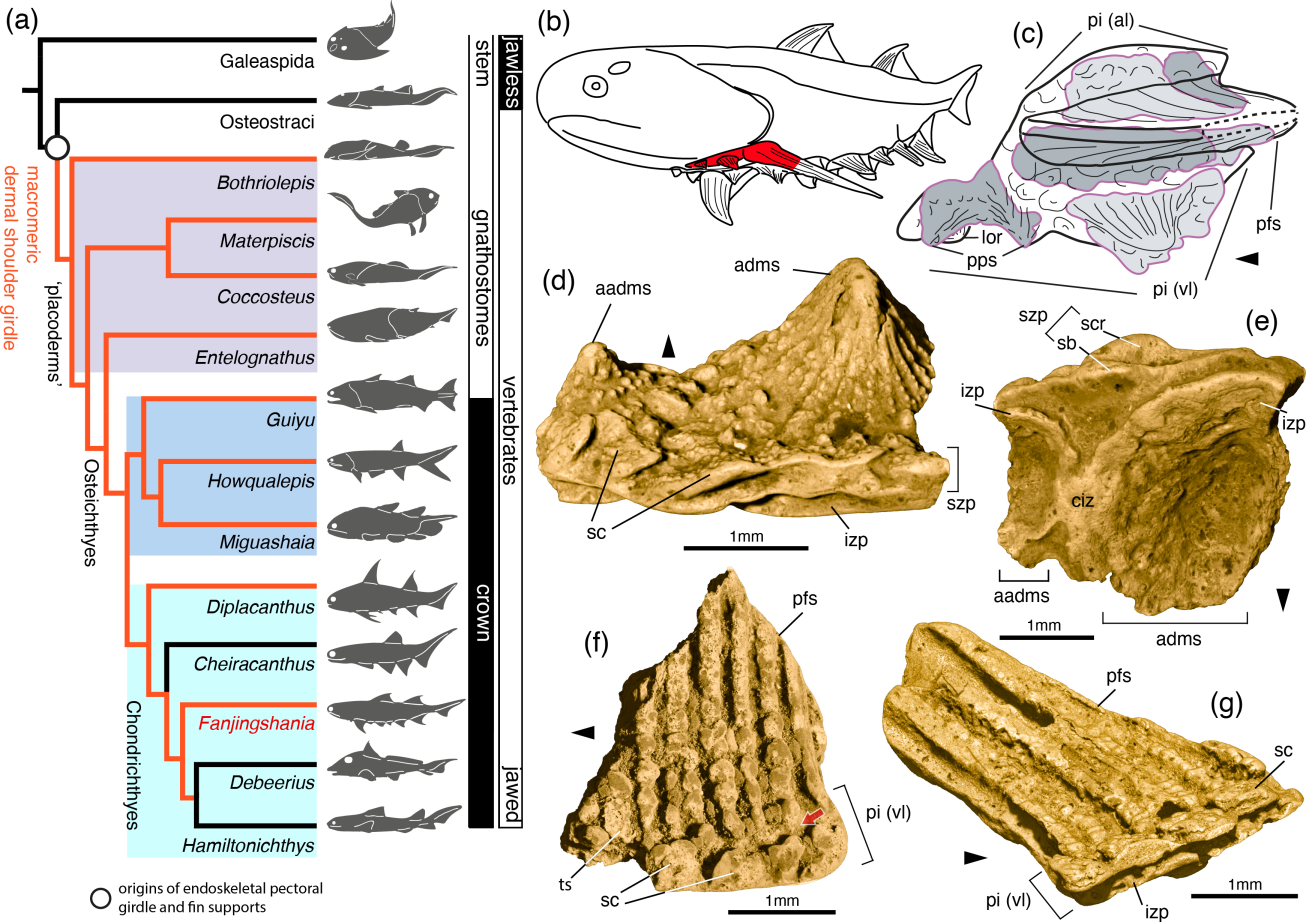
The shoulder skeleton of *F. renovata* in the context of early vertebrates. Diagram and line art (a*–*c), volume renderings of tomography data (d*–*g). (a) Homology hypothesis for the pectoral girdle and appendicular skeleton of jawed vertebrates, data from this study and [1,2]. Simplified 50% majority-rule phylogenetic tree of early vertebrates (see electronic supplementary material). (b) Life reconstruction of *Fanjingshania* (the region in red is shown in c). (c) Lateral view reconstruction of the left pectoral spine-plate complex of *F. renovata* highlighting the position of studied specimens. (d) Posterior and (e) external views of a pair of admedian spines fused to an incomplete pinnal plate (IVPP V27434.2). (f) External (IVPP V27433.11) and (g) external lateral (IVPP V27433.12) views of fragments of pinnal plates fused to incomplete walls of pectoral fin spines. aadms, accessory admedian spine; adms, admedian spine; ciz, contact surface of the inner zone extensions of the prepectoral spines; izp, inner zone of the pinnal plate; lor, lorical plate; pfs, pectoral fin spine; pi (al), ascending lamina of the pinnal plate; pi (vl), ventral lamina of the pinnal plate; pps, prepectoral spine; sb, scale base; sc, body-type scale; scr, scale crown; szp, superficial zone of the pinnal plate; ts, tuberculate scale. Black arrowheads point towards the anterior and red arrows indicate cement line surfaces.

The fossil record [1–3] provides evidence for a stepwise acquisition of an endoskeletal pectoral girdle and associated paired fins at the transition from jawless to jawed vertebrates (figure 1a), although the sequence of these evolutionary events is still debated [1]. Paired fin-like extensions of the body wall [3,4] originate in stem gnathostomes (vertebrates capable of skeletal mineralization) and in derived jawless groups they can be supported by a cartilaginous skeleton [3–5] (figure 1a) attached to the cephalothorax. In jawed vertebrates, a differentiation of a shoulder girdle independent of the head region coincided with the evolution of a pectoral dermal skeleton of large (macromeric) plates that integrate tightly with the elements of the scapulocoracoid [1,6,7] (figure 1a). A common feature associated with the complex of ventral (coracoid) and dorsal (scapular) pectoral plates of jawed stem gnathostome (‘placoderm’) lineages is a pair of fin spines [6,8]. Of the two major divisions of modern jawed vertebrates (osteichthyans and chondrichthyans), pectoral fin spines have a limited distribution in early osteichthyans [9,10], but represent a substantial component of the shoulder skeleton of stem chondrichthyan (‘acanthodian’) groups [11] (electronic supplementary material, figure S5). Uniquely among jawed vertebrates, a subset of stem chondrichthyans possess an array of paired prepectoral and admedian spines that fuse together with pectoral plates and fin spines into a continuous dermoskeletal unit [11–15]. The homology of this spine–plate complex with the dermal shoulder elements of ‘placoderms’ and early osteichthyans has been questioned [16,17] and reflects difficulties in discriminating between pectoral spines and spine-shaped plates in fossil groups. In living vertebrates dermoskeletal elements develop from mesenchymal condensations (papillae) and exhibit ontogenetic differences in the organization of the papillae and surrounding collagen matrix between bony plates and fin spines [18–21]. Nevertheless, in the absence of neontological data on bone morphogenesis outside osteichthyan/tetrapod models [22], the dermal shoulder ‘armour’ of ‘acanthodians’ from the lower Silurian–Upper Devonian (c. 440−370 Mya) [11] is central to understanding how chondrichthyans formed macromeric ossifications.

The available histological information on the dermal skeleton of stem chondrichthyans identifies their pectoral plates and fin spines as multilayered composites of odontogenically derived surface sculpture and inner osteogenic tissues [11–13,23,24]. Despite that, surprisingly little is known about the growth of the dermal pectoral girdle of early chondrichthyans, particularly in taxa where its components fuse into a continuous structure (e.g. in *Dobunnacanthus* [15]*, Ptomacanthus* [25]*, Climatius* [26] and *Diplacanthus* [12]). Three-dimensional imaging has proven crucial in reconstructing complex developmental sequences in the skeleton of early vertebrates [27–30], although these techniques have not been applied to the shoulder girdle of stem chondrichthyans beyond anatomical investigations [25,31].

Here we use high-fidelity synchrotron radiation X-ray tomographic microscopy to study the growth of the dermal skeleton of the *ca* 439-million-year-old chondrichthyan *Fanjingshania renovata* [24] (figure 1b,c), the stratigraphically oldest jawed vertebrate preserving a pectoral girdle. The new data allowed us to establish a developmental model for the pectoral girdle and spine complex of stem chondrichthyans and link it to the processes of osteogenesis and odontogenesis in modern vertebrates. We further investigated the evolutionary implications of the results on the perceived homology of macromeric dermal bone across the gnathostome clade.

## 2. Material and methods

### (a) Material

The studied skeletal elements of *F. renovata* are part of a larger fossil collection (more than 1000 fin spines, spines, tesserae, scales and dermal plates belonging to the species) recovered from the lower Silurian (Aeronian) beds of the Rongxi Formation, exposed near the village of Leijiatun, Shiqian county, Guizhou province, China [24]. They are represented by a pinnal plate fragment (IVPP V27433.8) and other incomplete pinnal plates fused to prepectoral (IVPP V27433.9) and admedian (IVPP V27434.2) spines and pectoral fin spine fragments (IVPP V27433.10, IVPP V27433.11, IVPP V27433.12, IVPP V27433.4). All specimens were deposited at the Institute of Vertebrate Paleontology and Paleoanthropology (IVPP), Chinese Academy of Sciences.

### (b) Synchrotron radiation X-ray tomographic microscopy

The fossil material was analysed with synchrotron radiation X-ray tomographic microscopy at the TOMCAT (X02DA) beamline of the Swiss Light Source, Paul Scherrer Institute, Villigen, Switzerland. The scans were performed with a 10× and a 4× objectives, producing 1501 and 1201 projections, respectively, acquired over a 180° rotation arc. The beam energy was set to either 25 or 28 keV and the beam was attenuated with 100 μm Al, 10 μm Cu and 10 μm Fe filters. The projections were processed with tomographic reconstruction algorithms developed by Marone *et al.* [32], which produced tomographic slices with pixel size of 1.625 μm (4× objective) and 0.65 μm (10× objective).

Analysis, segmentation and 3D visualization of the tomographic slices was performed in Dragonfly software, version 2022.2 (www.theobjects.com/dragonfly).

### (c) Phylogeny

We performed a phylogenetic analysis under parsimony criteria based on a data matrix modified from Brazeau *et al.* [1] (electronic supplementary material, table S1). The matrix was analysed in TNT version 1.6 [33] employing the traditional search function. Search parameters were set to random seed value of 2, number of addition-sequence replicates to 1000, a tree bisection reconnection (TBR) swapping algorithm limited to 100 trees per replication and a tree buffer of 10 000. The analysis was constrained by designating the galeaspid *Hanyangaspis guodingshanensis* as an outgroup taxon and the Osteostraci as monophyletic according to the current phylogenetic framework of early vertebrates [1,2]. This produced 1800 most parsimonious trees (MPTs) with a length of 1198 steps.

Bayesian analysis was also conducted for the same data matrix in MrBayes version 3.2.7. We ran a Markov chain Monte Carlo (MCMC) simulation for 10 million generations with *Hanyangaspis guodingshanensis* set as an outgroup and jawed vertebrates constrained as monophyletic. Burn-in fraction was set to 25%, the sampling frequency of the chain to 1000 generations and the number of simultaneous MCMC chains to four.

Ancestral state reconstructions were carried out for the parsimony and Bayesian trees (for details, see the electronic supplementary material).

## 3. Description

### (a) External features

**Figure 2.**
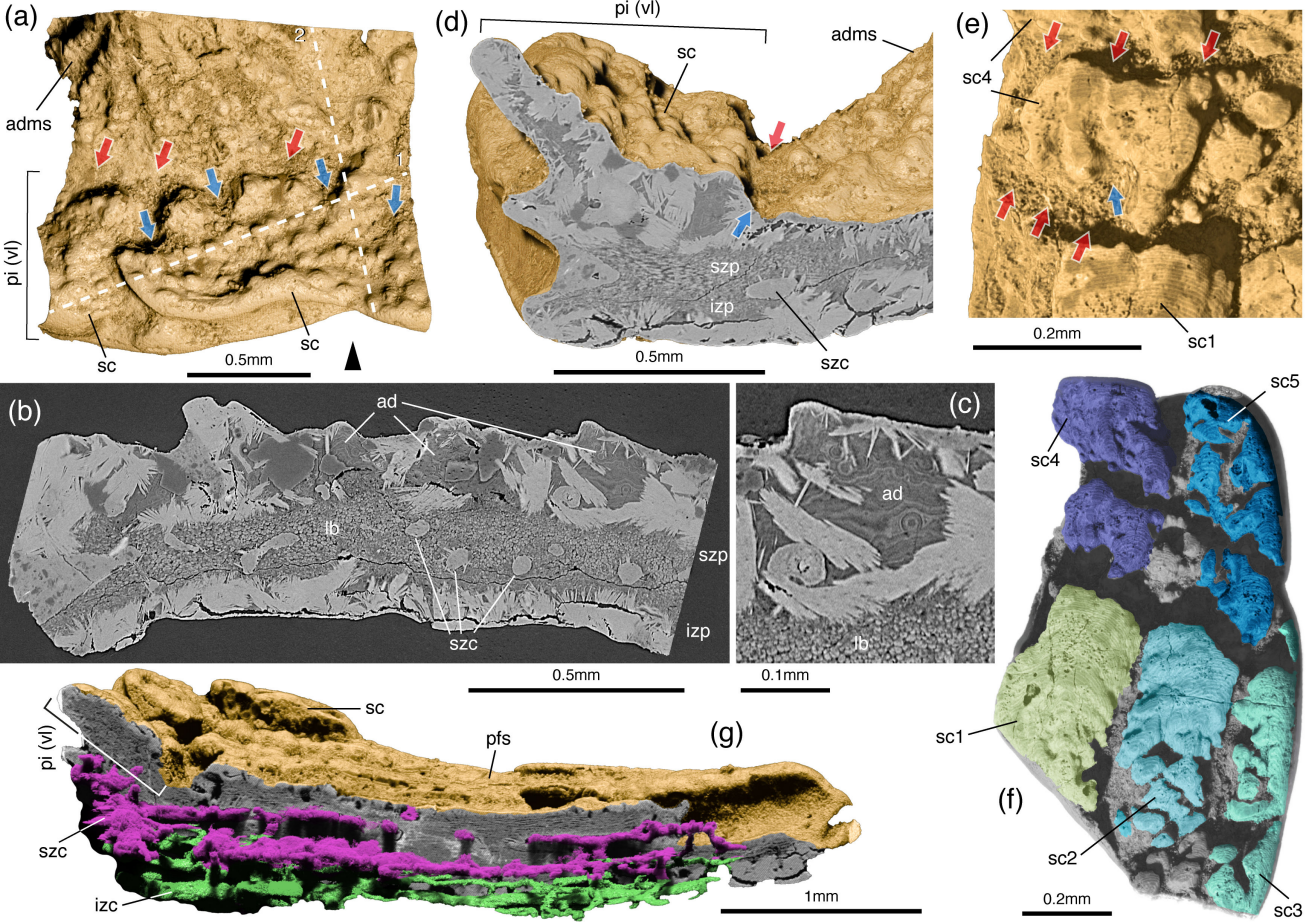
Resorption and remodelling features and internal structure of the dermal shoulder girdle of *F. renovata*. Volume renderings of tomography data (a, d–h) and tomography slices (b, c). (a) External view of (IVPP V27434.2) showing a portion of the admedian spine and the pinnal plate. (b) Virtual slice across the pinnal plate of IVPP V27434.2 along plane 1. (c) Detail of (b) showing the structure of the atubular dentine of the pinnal scales. (d) Virtual slice through IVPP V27434.2 along plane 2 depicting the structure of the pinnal plate at the junction with the admedian spine. (e) Detail of partially resorbed scales of the pinnal plate fragment (IVPP V27433.8 shown in (f)). (g) Longitudinal virtual slice of IVPP V27433.12 revealing the canal network inside the pectoral fin spine and the pinnal plate. ad, atubular dentine; adms, admedian spine; izc, canals of the inner zone; izp, inner zone of the pinnal plate; lb, lamellar bone; pfs, pectoral fin spine; pi (vl), ventral lamina of the pinnal plate; sc, body-type scale; szc, canals of the superficial zone; szp, superficial zone of the pinnal plate. Black arrowhead points towards the anterior, red arrows indicate cement line surfaces and blue arrows indicate resorption surfaces.

In the examined material, fragments of dermal ventral (pinnal) plates are fused to partial or complete spines (figures 1b–g and 2, electronic supplementary material, figure S1) that together represent prepectoral, admedian and pectoral fin spines associated with the shoulder girdle of *Fanjingshania* [24]. These preserve as pairs of differently sized prepectoral (figure 2f, electronic supplementary material, figure S1a,b) and admedian (figure 1d,e) spines and side wall remnants of pectoral fin spines (figure 1f,g, electronic supplementary material, figure S1) identified by the irregular spacing and thickness of their ornamenting ridges. At the base of the spines the ridges fragment into strings of connected and/or independent nodes that reach the level of the pinnal plate and/or are clearly separated from it by an excavated, unsculptured area (figures 1f,2 and 2a,d).

Modified body scales represent a major structural component of the superficial zone of the pinnal plates. Individual scales cannot be recognized only in the parts of the pinnal plate formed immediately around the prepectoral spines in IVPP V27433.9 (electronic supplementary material, figure S1a) and between the admedian spines (figure 1d, electronic supplementary material, figure S1c) where the plate exhibits a nodular sculpture.

Conspicuous resorption features cut through the pinnal plate scales adjacent to the bases of pectoral and admedian fin spines. They manifest as linear grooves, crescent-like pits or larger resorption fields that create jagged crown margins and fragment the scale into clusters of tubercles (figure 2a,d–f*,* electronic supplementary material, figure S2a,e). The orientation of the linear resorption features is variable and can be parallel, perpendicular or even oblique to the long axis of the scales.

The bases of the pinnal scales are low and fuse together into a single structure best observed at the periphery of the specimens where they protrude from the recessed/eroded margin of the inner zone of the plate (figure 1d,e,g). The latter transitions without a break from the inner spine wall and in IVPP V27433.9 and IVPP V27434.2 its margin is continuous with a series of elliptical growth increments that originate from the admedian and prepectoral spines (figure 1e, electronic supplementary material, figure S1b). Wedge-shaped recesses in the inner pinnal plate flank a thickened area of contact/fusion between the pinnal plate extensions of the two prepectoral and admedian spines (figure 1e, electronic supplementary material, figure S1b).

### (b) Internal features

The primary tissue at the base of the shoulder girdle spines is lamellar bone (vascular zone of [24]) with fibre bundle orientation that is parallel to the spine surface (figure 2b–d,g, electronic supplementary material, S2b–e*,* S3c–e). In the discontinuous ridges of the pectoral fin spines adjacent to the pinnal plate, the bone is substituted by atubular dentine with extensive globular mineralization (electronic supplementary material, figure S2c) that contrasts with the tubular dentine varieties reported in stratigraphically younger ‘acanthodians’ [11]. The histology of the ornament of the prepectoral and admedian spines is not discernible due to recrystallization. A diagenetic texture is also observed in the conical core of the anterior prepectoral and the admedian spines (electronic supplementary material, figure S2g). This structure corresponds in topology to the globular calcified cartilage that infills the central cavity of the pectoral fin spines [24] but the absence of histological detail prevents its identification.

The fibre bundles of the pinnal bone are organized similar to those of the spines, but in the superficial zone they change orientation by bending upwards immediately below the scales of the plate (figure 2d). In virtual sections, the pinnal scales reveal bony pyramidal bases and compound multi-element (polyodontode) crowns akin to those of typical body scales (electronic supplementary material, figure S2e,f). Despite extensive diagenetic recrystallization of the scale crowns, isolated patches of their original tissue were detected in the high resolution (submicron) synchrotron tomographic data. The crown tissue is indistinguishable from the atubular dentine of the discontinuous ridges of the spines and shows incremental depositional waves formed concentrically around vestiges of pulp spaces as well as sporadic globular mineralization (figure 2b–d).

Interruption of the scale structure by resorption surfaces occurs in the crown dentine and the basal bone and manifests as sloped or scoop-shaped surfaces cutting across multiple odontode generations or the periphery of the base (figure 2d–f, electronic supplementary material, figure S2a,e,f).

Some of the specimens (figure 1f*,* electronic supplementary material, figure S2a*–*c) demonstrate partially resorbed scales with conspicuous tuberculate ornament that differs from the typical ridged sculpture of pinnal scales. These tuberculate elements represent the modified primordial region of fragmented pinnal scales, whose appearance is given by closely packed stubby odontodes instead of the row of flattened deltoid odontodes possessed by complete pinnal scales (electronic supplementary material, figure S2a,b,f).

The pectoral fin spine wall of IVPP V27433.12 (figure 2g, electronic supplementary material, figure S2h) preserves details of a vascular system that features interconnected longitudinal canals developed inside the superficial and inner zones of the spine. The superficial canals give rise to mesh canals located inside the [34] spines’ ornament where they open on the lateral sides of individual ridges. In the prepectoral and admedian spines, remnants of canals are detected at the boundary between the superficial and inner zones of the spine wall (electronic supplementary material, figure S2g,i), which run parallel to the ornamenting ridges in direction of the spine apex.

The tiered system of canals of the pectoral spines extends into the pinnal plate but does not enter the basal bone of the pinnal scales. Instead, it connects to a deep set of V-shaped canals housed inside the thickened area of the plate that exists at the contact between the extended inner walls of prepectoral (electronic supplementary material, figure S2g,i) and admedian spines.

## 4. Discussion

### (a) Growth mechanics

The plate-spine shoulder complex o*f Fanjingshania* displays resorption and reversal (cement line) features indicative of periodic remodelling of dermal bone and dentine at the base perimeter of the pectoral spines. These mark areas of circumferential growth in the spines, which occurs at the transition with the pinnal plate and along the contact between the inner bone tissue of adjacent spines (electronic supplementary material, figure S4a).

**Figure 3.**
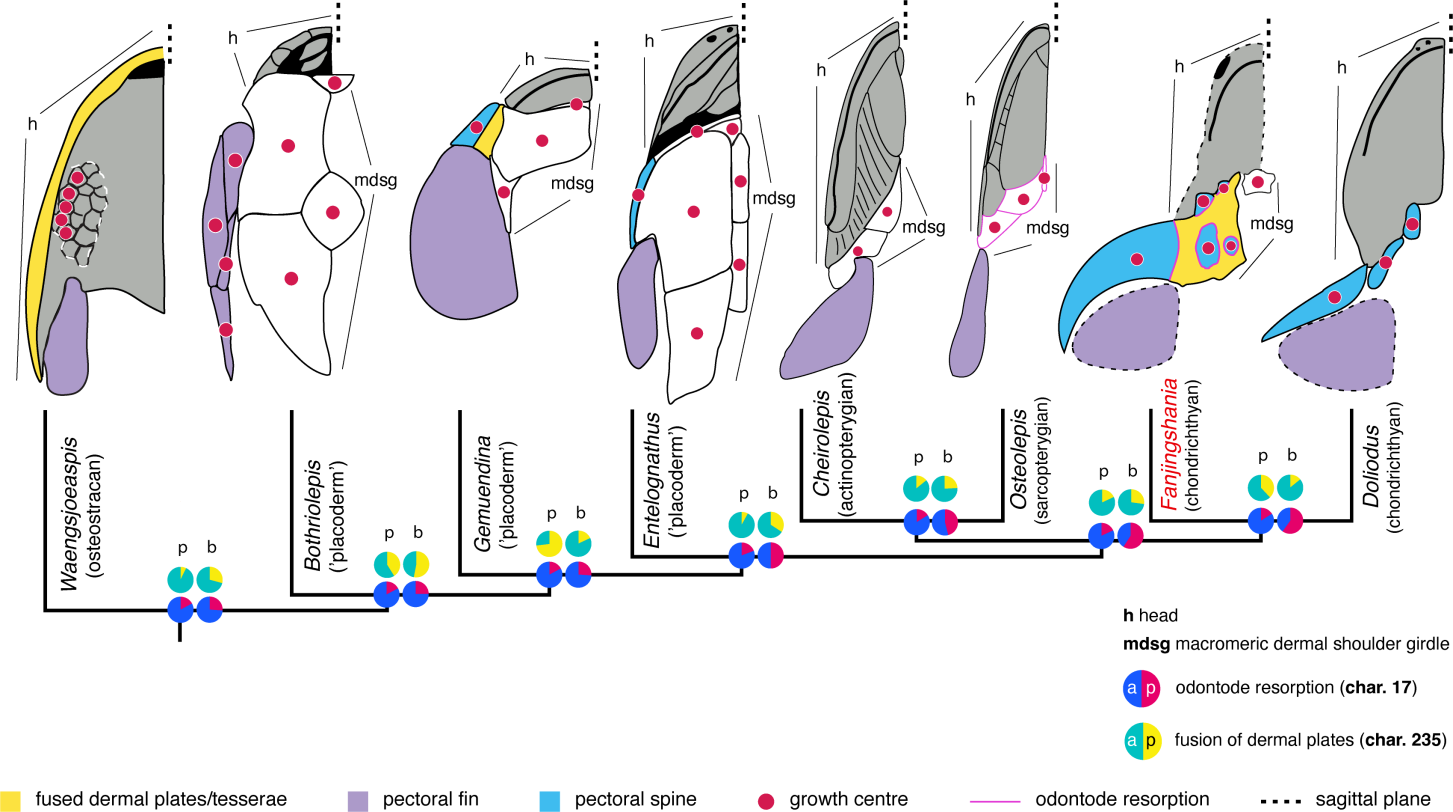
Evolution of growth-related characters in the dermal shoulder girdle of *F. renovata* mapped onto a summary of parsimony and Bayesian phylogenies of early vertebrates. Information for the reconstruction of ancestral state characters and the phylogenetic analysis is available in the electronic supplementary material. Diagrams show the right half of the head and shoulder regions and are based on the current and previous studies (see electronic supplementary material). Character numbers correspond to their position in the data matrix. Pie chart letters in legend stand for character (a) absence and (p) presence. Ancestral state probabilities for (p) parsimony and (b) Bayesian analyses.

The growth of the pectoral spines is cyclical and involves extension of the spines’ bony ridges and an accompanying resorption of the neighbouring scales of the pinnal plate. This process can be subdivided into hypothetical steps (electronic supplementary material, figure S4b) that are similar to the resorption, reversal and formation phases of the endochondral bone remodelling cycle [35,36] in humans and mammalian model species. Removal of the pinnal scales requires multiple resorption events that cause the systematic reduction of the scale crown surface. The transition to a formation phase is associated with the presence of reversal surfaces [37] (surface grooves, figure 2a,d–f) separating the spine ornament from the pinnal plate. In endochondral bone [36,38] these reversal surfaces are prerequisite for new bone deposition and similarly in *Fanjingshania* they are suggested to demarcate the boundary of active growth of the spines’ dermal bone ornament. We infer that the areas of fusion between the spines also undergo periodic bone resorption (figure 3a) as this would be essential to create space for the expansion of the inner zone of the spines. Although the original bone structure at these sites is largely obscured by recrystallization, their extensive vascularization (electronic supplementary material, figures S2g,l*,* S3a,b) presents indirect evidence for the remodelling of the tissue through open osteonal canals [39].

Individual pinnal plates in *Fanjingshania* are a product of the five separate pectoral spines associated with each half of the shoulder girdle. A peculiar aspect of the plates’ development is that their enlargement, and that of the spines, involves the incorporation and subsequent resorption of body scales. Examples where the ontogeny of fin spines features the removal of adjacent elements of the dermal skeleton have not been previously documented in extinct [40,41] or modern gnathostomes [19,42]. Some parallels can be drawn, however, with the morphogenesis of the shoulder girdle endoskeleton of teleosts [42,43], which is achieved by subdivision of a cartilaginous precursor plate into scapular, coracoid and radial elements and their ossification. Although the pinnal scales of *Fanjingshania* are similarly subjected to fragmentation they do not serve as precursors for the differentiation of the components of the dermal shoulder girdle. The substitution of dentine by bone (or the reverse) recognized in *Fanjingshania* has been recorded in the context of disease- and injury-induced tissue replacement [44–46] in the oral and extra-oral dermal skeleton. Nevertheless, we regard this remodelling process to be physiological in origin due to its occurrence in a variety of specimens and its inferred cyclical nature.

### (b) The macromeric shoulder girdle of stem chondrichthyans

The spine-derived bony plates of *Fanjingshania* supply evidence for a previously undocumented assembly of growth processes in the dermal skeleton of early vertebrates. Our data suggest that *Fanjingshania*’s compound plates may provide a general model for macromery in chondrichthyans. Indirect proof for this comes from the reported fusion of body scales into pinnal plates and the integration of these compound structures into a single spine–plate complex among climatiid and diplacanthid stem chondrichthyans [12–15]. Significantly, a reduction or absence of the macromeric shoulder girdle does not appear to be associated with a corresponding loss of pectoral spines in early chondrichthyans [11,47] (figure 3). Coupled with the data from *Fanjingshania* this reinforces our view of the pinnal plates as part of the growing margins of pectoral spines, with the latter likely regulating the extent of the plates throughout ontogeny.

The formation of dermal plates in *Fanjingshania* is also contingent on periodic resorption of bone and dentine around the margins of individual growth zones. Recognizing this capacity in the chondrichthyan stem group implies that it is a plesiomorphic trait of crown gnathostomes, as predicted by our Bayesian phylogeny (figure 3). Competence for remodelling the other major skeletal system, the endoskeleton, appears to be primitively absent in chondrichthyans [48,49] in contrast to osteichthyans [42,50]. This alludes to similarities between chondrichthyans and the plesiomorphic for jawed vertebrates conditions demonstrated by ‘placoderms’, where the processes of active remodelling are confined to the dermal skeleton [51–54].

Fusion of dermoskeletal elements during ontogeny satisfies the predictions of the classical odontode regulation theory [55] for the initial state of macromery. Our results nevertheless establish a reverse evolutionary scenario where stem chondrichthyans represent a transitional stage from the macromeric skeleton of stem gnathostomes to the micromeric conditions of crown chondrichthyans. The division of the dermal skeleton into strictly micro- or macro-meric categories [7,22] is questioned here given the occurrence of compound bony plates in *Fanjingshania* as well as in the headshield of jawless stem gnathostomes [56,57] and in a subset of ‘placoderms’ [6] (figure 3). Instead, we propose that the chondrichthyan shoulder girdle possesses distinct histological properties related to a cryptic developmental diversity of macromeric conditions, which can inform competing hypotheses for the homology of these ossifications in jawed vertebrates [7].

## Data Availability

Data and code files are deposited in the Dryad Digital Repository [58]. Supplementary material is available online [59].
